# DEVELOPING A NEW FEDERAL POLICY AGENDA TO ADDRESS SOCIAL ISOLATION AND LONELINESS

**DOI:** 10.1093/geroni/igz038.2234

**Published:** 2019-11-08

**Authors:** Andrew MacPherson

**Affiliations:** Coalition to End Social Isolation & Loneliness, Washington, District of Columbia, United States

## Abstract

The Coalition to End Social Isolation and Loneliness convenes a diverse group of allied stakeholders, including consumer and patient groups, health plans, community-based organizations, private sector researchers and innovators, and others, to address the epidemic of social isolation and loneliness. The Coalition is developing and advocating for bipartisan federal policy solutions to provide individuals the support they need to be socially engaged. This session will describe the role of the Coalition in engaging stakeholders, promoting innovative research, and advocating for policy changes that combat the adverse consequences of social isolation and loneliness and advance approaches and practices that improve social connectedness for all Americans.

